# Wearing compression garments differently affects monopodal postural balance in high-level athletes

**DOI:** 10.1038/s41598-020-72347-2

**Published:** 2020-09-18

**Authors:** Kévin Baige, Frédéric Noé, Thierry Paillard

**Affiliations:** Laboratoire Mouvement, Equilibre, Performance et Santé (EA 4445), Université de Pau et des Pays de L’Adour/E2S UPPA, ZA Bastillac Sud, 11 rue Morane Saulnier, 65000 Tarbes, France

**Keywords:** Motor control, Sensorimotor processing

## Abstract

This study investigated the acute effects of compression garments (CG) on balance control in elite athletes. 15 male professional handball players were recruited. They had to stand as motionless as possible in a monopedal stance on a force plate with the eyes closed and on a wobble board with the eyes open, while wearing CG or not. Centre of foot pressure mean velocity and surface area were calculated. Statistics were first calculated with the data from the whole sample. A hierarchical cluster analysis was also performed in order to categorize the participants’ behaviours into subgroups with similar characteristics. The whole group analysis showed that there were no significant effects attributed to CG. The clustering analysis identified two distinct and homogeneous subgroups of participants. Only athletes with the best balance abilities at baseline could benefit from CG wearing to improve their balance control. These athletes, who swayed less and were more sensitive to somatosensory manipulation due to CG wearing, seem to control balance by adopting a support-dependent preferred sensorimotor tactic. Our findings suggest that amongst high-level athletes, the ability to benefit from CG wearing to improve balance control seems to depend on participants’ intrinsic balance skills and/or preferred sensorimotor tactics.

## Introduction

Compression garments (CG) have been widely used in medicine in patients with venous disease, restricted mobility or with an immobilization of the lower limb for many years^1^. CG create an external pressure gradient on the body surface which improves venous hemodynamics and prevents leg swelling and blood clots^1,2^. The wearing of CG in sport has also become very popular during and after training or competition in order to improve physical performances^3–5^ and to accelerate recovery^6,7^. Suggested mechanisms include attenuation of muscle oscillation during exercise^4,5^, enhanced venous return, blood flow and muscle oxygenation with accelerated metabolic removal^2,8^, reduced post-exercise edema^9^ and attenuated markers of muscle damage^6^. CG could have a beneficial influence on endurance exercise mainly by attenuating perceived exertion and muscle soreness and by improving running economy^4,5^. CG could also affect neuromuscular performances such as vertical jumping and sprint performance. The exact mechanisms by which CG impact neuromuscular performances are not clear, but it may be due to the activation of cutaneous mechanoreceptors which elicits enhancements in the ability to perceive position in space and improves accuracy of motor actions such as jumping technique^4,10,11^. The additional sensory cues from cutaneous mechanoreceptors provided by CG are also likely to improve movement control such as balance control^12–16^.

When specifically focusing on the influence of CG on balance control, studies showed that CG did not bring any beneficial effect^17–22^, whereas others showed that balance control was enhanced when wearing CG^12,13,15,23,24^. The heterogeneity in these results might stem from participants characteristics (e.g., age, sex, healthy vs injured), conditions of balance control assessment (e.g. static vs dynamic postural tasks, monopodal vs bipodal stances, with eyes open or closed, on stable ground or unstable/compliant surface) and CG properties (segments/joints covered by the CG, level of compression). In general, CG rather induce beneficial effects when standing in monopodal stance^12,13,23,24^, especially with the eyes closed^12,13,24^. Only Woo et al. reported a positive influence of CG with a bipedal stance when assessing elderly participants who had to perform a Romberg test on a compliant surface^15^. CG seem to preferentially benefit to subjects with lower limb injuries^12,23,24^ than to non-injured subjects^17–20^. The location of the compression (i.e. ankle, shank, knee or thigh) does not seem to be a major factor in explaining the differences observed in the studies. Some studies reported beneficial effects of CG on balance control with knee sleeves^12,23^, socks^15^ and leggings^13^ whereas others did not report any beneficial effect with knee sleeves^17^ and socks^18–20,22^. Similarly, the level of compression, which is not systematically specified in the studies, does not appear to be a major factor in explaining the heterogeneity of the results. Studies that have tested the effects of wearing CG of different compression levels reported that the level of compression had no influence on balance control^15,19–22^.

Overall, CG would rather improve balance control in physically diminished/impaired subjects (e.g., elderly and injured) than in healthy young ones in particular in monopedal stance. Nonetheless, knowing that expertise in sport can enhance the process of sensory reweighting^25^, expert athletes are likely to integrate more efficiently the cutaneous stimulation provided by CG to improve balance control. Only two studies, to our knowledge, have been conducted in order to test the influence of CG on balance control in sportspeople, while producing divergent findings^13,22^. Michael et al. showed that balance control of sportswomen was improved when they wore CG^13^, whereas Sperlich et al. did not report any beneficial influence of CG in a group of competitive alpine skiers^22^. Nevertheless, Sperlich et al. performed a balance test with and without CG while asking participants to wear racing ski-boots^22^. These boots, which stimulate cutaneous mechanoreceptors in the foot and shank and provide a mechanically efficient support at the tibia level^26^, might have acted as a confounding factor and hid a potential positive effect of CG. Hence the influence of expertise in sport on the ability to take advantage for CG to improve balance control remains an open question.

Consequently, this study was undertaken in order to investigate the acute effects of calf compression sleeves on monopodal balance control of professional elite handball players. It was hypothesized that calf compression sleeves would enhance monopodal balance control in professional handball players. Because of potential heterogeneity between participants’ response to CG wearing, a normalized principal component analysis was used to reduce the dimensionality of the data before performing a hierarchical cluster analysis (HCA). The HCA is a multivariate statistical method which categorizes the participants’ behaviours into subgroups with similar characteristics, thus facilitating the investigation of differences in individual responses by enabling the identification of natural groupings that may exist in a whole sample^27,28^.

## Results

Table 1 presents the mean values of COP parameters in the whole sample with the characteristics of each cluster. When inferential statistics were performed on the whole group, no significant differences were observed between REF and CG conditions in the STA postural task. Nevertheless, strong tendencies could be observed on VX and VY, which tended to be reduced in CG compared to the REF condition (V = 92; *P* = 0.073 and V = 91; *P* = 0.083). In the UNSTA postural task, no statistically significant differences or tendencies were observed between both conditions.

**Table 1:** COP parameters of the whole group with cluster characteristics in the different postural tasks and experimental conditions.

			n	REF	CG	RD
STA	S	Whole group	15	1,978.2 (536.3)	1,822.4 (736.7)	− 7.9 (25.1)
		Cluster 1	8	1,725.8 (443.3)	1,281.3 (301.2)	− 22.1 (22.6)
		Cluster 2	7	2,266.8 (509.8)	2,440.8 (569.1)	8.5 (17.0)
	VX	Whole group	15	59.4 (15.2)	54.8 (15.0) ^§^	− 7.2 (16.0)
		Cluster 1	8	52.3 (4.4)	44.9 (8.5)	− 14.2 (14.2)
		Cluster 2	7	67.5 (19.3)	66.1 (12.9)	0.7 (15.1)
	VY	Whole group	15	46.7 (13.8)	43.1 (17.5) ^§^	− 9.7 (18.8)
		Cluster 1	8	38.7 (4.4)	30.0 (5.9)	− 22.6 (12.7)
		Cluster 2	7	55.9 (15.3)	58.0 (13.5)	5.0 (12.7)
UNSTA	S	Whole group	15	707.0 (206.9)	648.7 (369.3)	− 9.7 (38.8)
		Cluster 1	8	610.3 (130.6)	481.7 (201.2)	− 21.3 (28.1)
		Cluster 2	7	817.5 (230.7)	839.6 (437.4)	3.5 (47.0)
	VX	Whole group	15	31.8 (12.2)	34.5 (17.7)	5.5 (16.9)
		Cluster 1	8	23.2 (6.6)	22.2 (5.1)	− 2.9 (13.5)
		Cluster 2	7	41.6 (9.4)	48.6 (16.4)	15.1 (15.9)
	VY	Whole group	15	23.4 (7.1)	22.3 (7.7)	− 5.3 (12.8)
		Cluster 1	8	18.5 (5.1)	16.1 (3.8)	− 11.9 (8.9)
		Cluster 2	7	29.0 (4.2)	29.4 (3.5)	2.2 (12.9)

Data are expressed as mean (SD).

*STA* stable postural task with the eyes closed, *UNSTA* unstable postural task with the eyes open, *REF* reference condition, *CG* compression garments condition, *S* COP surface area, *VX and VY* mean COP velocity along the medio-lateral and antero-posterior axes respectively, *RD* relative difference between both conditions (RD = 100*[CG-REF]/REF).

^§^illustrates a tendency from whole group statistics (0.05 ≤ *P* < 0.1) between REF and CG conditions.

**Figure 1:**
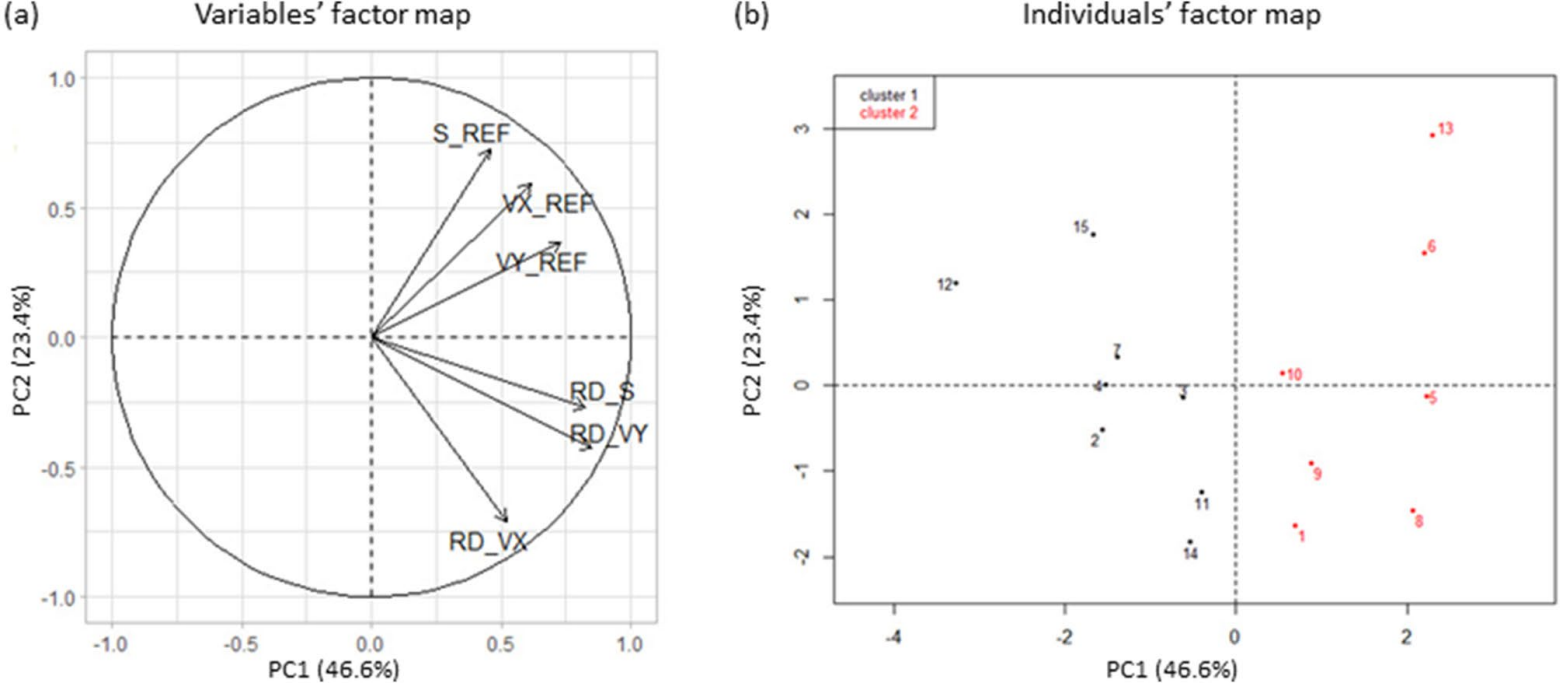
Variables’ (**a**) and individuals’ (**b**) factor map of the PCA applied on COP parameters in the STA postural task. Individuals from cluster 1 and 2 are represented by black and red dots respectively. *S_REF* COP surface area in the REF condition, *VX_REF* mean COP velocity along the medio-lateral axis in the REF condition, *VY_REF* mean COP velocity along the antero-posterior axis in the REF condition, *RD_S, RD_VX and RD_VY* relative difference between both conditions (RD = 100*[CG-REF]/REF) of S, VX and VY variables.

**Figure 2:**
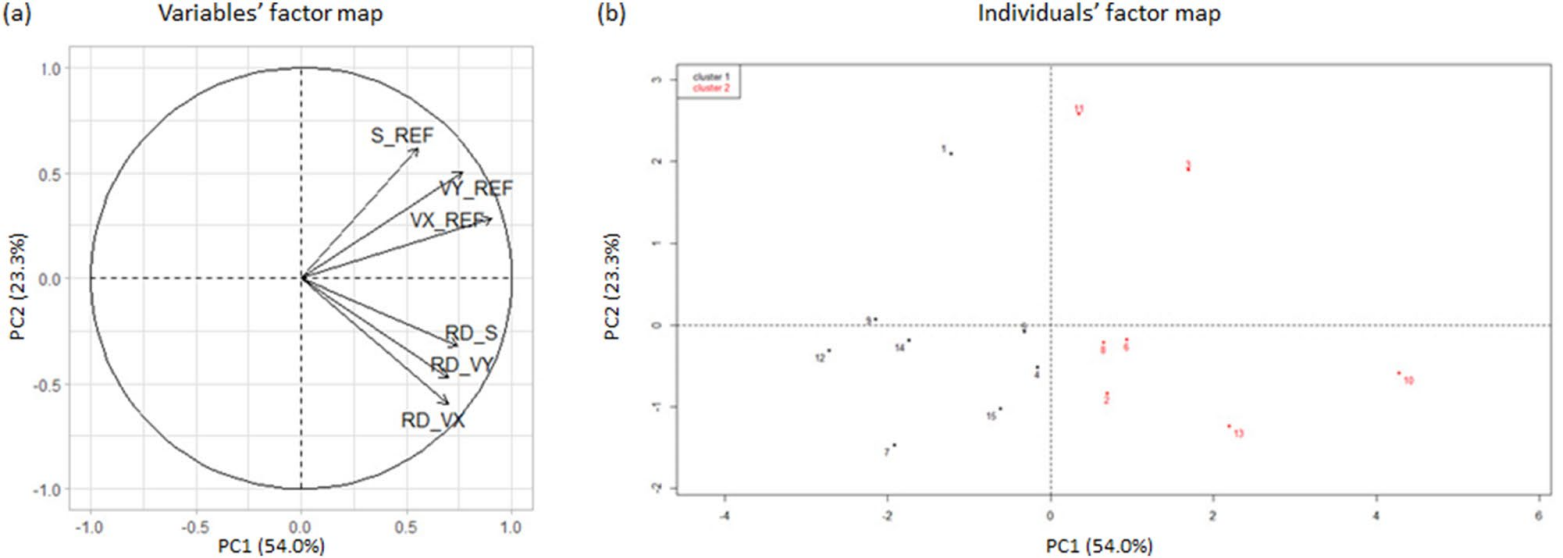
Variables’ (**a**) and individuals’ (**b**) factor map of the PCA applied on COP parameters in the UNSTA postural task. Individuals from cluster 1 and 2 are represented by black and red dots respectively. *S_REF* COP surface area in the REF condition, *VX_REF* mean COP velocity along the medio-lateral axis in the REF condition, *VY_REF* mean COP velocity along the antero-posterior axis in the REF condition, *RD_S, RD_VX and RD_VY* relative difference between both conditions (RD = 100*[CG-REF]/REF) of S, VX and VY variables.

In the STA postural task, PCA resulted in two components that explained 70% of the total variance of the original dataset. PC1 accounted for 46.6% of the total variance and was loaded with three variables, RD_VY, RD_S and VY, which were positively correlated. PC2 explained 23.4% of the total variance and was loaded with three variables, S and VX, which were positively correlated, and RD_VX which was acting in the opposite direction (Fig. 1a). In the UNSTA postural task, PCA resulted in two components that explained 77.3% of the total variance of the original dataset. PC1 accounted for 54.0% of the total variance and was loaded with the three variables, VX, VY and RD_S, which were positively correlated. PC2 explained 23.3% of the total variance and was loaded with three variables, S and VY, which were positively correlated, and RD_VX which was acting in the opposite direction (Fig. 2a). In both postural tasks, the clustering analysis identified two clusters. The individuals’ factor map (Figs. 1b and 2b) shows that there was a great variability between subjects and that individuals from these two clusters were mainly differentiated on the PC1 axis. Individuals from cluster 1 were located on the left side, with low values of postural parameters in the REF condition and negative values of relative difference between both conditions (Table 1). On the contrary, individuals from cluster 2 were located on the right side and had high values of postural parameters in the REF condition and positive values of relative difference between both conditions.

## Discussion

The aim of this study was to analyze the acute effects of wearing CG on the monopodal balance control in elite professional handball players. When considering data from the whole sample, results from inferential statistics were not consistent with the hypothesis that CG would improve balance control, since the wearing of CG only induced tendencies to an improved monopedal balance control in the STA postural task. The HCA approach was successful in identifying two distinct and homogeneous subgroups of participants, thus illustrating that high inter-individual variability in the ability to benefit from CG wearing to improve balance control is present within a group of high-level athletes.

CG act by applying frictional forces to the skin, which direction and size are related to the body sway movements^13^. As initially observed in studies about the effects of passive tactile cues on balance control^29^, these forces activate both slow and fast-adapting cutaneous mechanoreceptors to provide additional sensory cues that are integrated by the central nervous system to reduce body sway when a sensory pathway critical for standing posture has been perturbed (e.g. when vision is occluded). The stimulation of cutaneous inputs from the lower leg induced by the wearing of CG can also depress the H-reflex by activating Ia inhibitory interneurons^16^. Because an early spinal reflex can provoke small reactive and uncontrolled movements that can be a source of postural instability^30^, there is a relationship between the amplitude of the H-reflex and that of the displacement of the COP: the lower the amplitude of the H-reflex, the lower the postural sway^30,31^. Even though the differences in COP parameters were not statistically significant among experimental conditions, the tendencies to an improved balance control with CG (*P* = 0.073 and *P* = 0.083 for VX and VY respectively) could reflect an efficient sensory reweighting mechanism^32^ with an up-weighting of the additional cutaneous information provided by CG to compensate for the lack of visual cues in the STA postural task. Indeed, garments that stimulate lower limbs cutaneous receptors (e.g. CG, braces and tapes) rather positively influence balance control when somatosensory information is altered by experimental manipulations^13,33,34^. These garments had more limited effects when standing on unstable supports (e.g. wobble board as in the present study) or performing dynamic tasks (e.g. Y balance test)^17,20^, likely due to the availability of visual cues and the increased contribution of vestibular and proprioceptive cues due to greater joint movements^35^ which might have limited an up-weighting of the additional somatosensory cues provided by CG.

Results from the PCA showed that there was a high variability between individuals' responses to CG wearing within a population of professional elite athletes. This heterogeneity among the ability of participants to benefit from CG wearing to improve balance control could explain the controversial results about the influence of compression garments on balance control in healthy young subjects^13,14,17,18,20,24^. In both STA and UNSTA postural tasks, the HCA also provided evidence that this variability did not present a random structure, but was structured in two clusters of participants with similar characteristics. Subjects from cluster 1, while presenting lower values of postural variables in the REF condition, exhibited a more efficient balance control at baseline than subjects from cluster 2. Interestingly, only subjects from cluster 1 benefited from CG wearing to improve their balance control, while presenting negative values of RD of COP parameters between CG and REF condition. Individuals from cluster 2 presented positive value of RD and did not benefit from CG wearing. This link between balance control efficiency at baseline and the ability to take advantage of CG to improve balance control can potentially stem from (1) differences in participant’s preferred sensorimotor tactics and/or (2) differences in participants’ intrinsic balance/proprioceptive abilities.

Individuals vary in the degree to which they weight sensory inputs to control their balance^32^. Earlier reports about sensory reweighting in balance control during sensory perturbations have provided evidence that subjects from a homogeneous sample could respond differently to sensory perturbations depending on preferred modes of spatial referencing^36–38^. Two main *preferred sensorimotor tactics* have been identified. Some subjects, called *support-dependent*, rely more on the exploitation of the geometry of the support surface as a reference frame by assigning a high weight to somatosensory cues from ankle–foot proprioceptive and plantar cutaneous mechanoreceptors. Other subjects, called *gravity-dependent*, rely more on gravitoinertial signals by assigning a high weight to kinetic-graviceptive vestibular cues and/or to proprioceptive cues stemming from joints and muscles with a higher position in the kinematic chain (i.e. hip, trunk and neck joint muscles)^36–38^. Isableu & Vuillerme showed that subjects who had to sway as little as possible on stable ground with the eyes closed differently regulated their balance according to their preferred sensorimotor tactics^36^. Support-dependent subjects swayed less than gravity-dependant subjects, by minimising angular variations of the ankle with respect to the support. On the other hand, gravity-dependent subjects exhibited larger postural sway while adopting a more exploratory behaviour in order to extract kinetic-graviceptive information more efficiently^36^. In the present study, individuals from cluster 1, who swayed less than those from cluster 2, had a typical profile of support-dependent subjects, while individuals from cluster 2 may be characterized as gravity-dependent subjects. Isableu & Vuillerme also showed that support-dependent subjects were more sensitive to somatosensory alteration of the feet-support than gravity-dependent ones^36^. In the same manner, individuals from cluster 1 exhibited a greater sensitivity to somatosensory manipulation by being the only participants to take advantage of CG to improve balance control, thus supporting the idea that they would present a support-dependent preferred sensorimotor tactic.

Even though athletes exhibit better balance control than non-athletes^25,39^, such a heterogeneity of balance regulation mechanisms within our group of athletes might also stem from individual natural predispositions that would persist despite years of training^40^. It is known that the contribution of proprioception in balance regulation is increased in athletes and that elite athletes have superior proprioceptive abilities than sub-elite or non-athletes^25,41^. Nevertheless, Han et al. showed that superior proprioceptive abilities in high-level athletes was not correlated with training experience but was rather constrained by genetically determined factors^41^. Hence, it can be hypothesised that athletes from cluster 1 had exceptional neurobiologically determined proprioceptive acuity that enabled them to benefit from CG wearing through optimal sensory reweighting. The ability to take advantage of garments that stimulate cutaneous receptors such as CG to improve balance control seems to describe a U-shaped relationship according to subjects’ balance and/or proprioceptive abilities. Beneficial effects are reported in individuals with poor proprioceptive acuity and/or balance^24,42^ and with individuals with good proprioceptive acuity and/or balance^13^, whereas no beneficial effects are reported in healthy non-athletes subjects with “ordinary” balance and/or proprioceptive acuity^17,18,20,24^.

A limitation of the study is that although CG were individually fitted by choosing proper sizing according to the individual calf size and height, we did not measure the interface pressure applied by the CG. According to the manufacturer’s information, the CG used in this study are expected to provide a 19 mmHg pressure level over the calf. Due to the sizing issue, however, it is possible that 19 mmHg is not always the pressure actually applied. For example, with a M size suitable for a 38–43 cm calf circumference range, the pressure exerted by the CG should be higher with a circumference of 43 cm compared to 38 cm. In this case, the more intense pressure on the skin may provide a greater cutaneous stimulus with a larger impact on balance control. The fact that a commercially manufactured CG does not provide a homogeneous compression level with the same size might modulate the effects of CG on balance control. Hence, in the present study, this could also have influenced the distribution of the individuals within the different clusters. Nevertheless, studies that have tested the effects of wearing CG of different compression levels reported that the level of compression had no influence on balance control^15,19–22^, thus suggesting that the level of compression is not the most crucial factor that modulates the effects of CG on balance control. However, future studies should be conducted to specifically examine the influence of compression heterogeneity actually provided by a CG model of a given size on balance control. Further experiments are also needed to assess athletes’ preferred sensorimotor tactics (through sensory perturbation paradigms) and intrinsic proprioceptive abilities in order to explore the influence of these two factors on the ability of athletes to take advantage of CG to improve balance control.

## Conclusions

This study showed that among a sample of professional handball players with more than 15 years’ experience, only athletes with the best balance abilities at baseline could benefit from CG wearing to improve their balance control. Hence, it can be suggested that the ability to benefit from CG wearing to improve balance control depends on elite athletes’ intrinsic balance abilities and/or preferred sensorimotor tactics. The present finding emphasizes the necessity to address the issue of inter-individual variability when studying the effects of CG on balance control.

## Methods

### Participants

Fifteen male handball players from the same team in the professional French National Handball League (age: 25.06 ± 4.19 years old, height: 190.5 ± 5.73 cm; body mass: 94.2 ± 12.25 kg; years of practice: 15.94 ± 5.15 years; mean ± SD) were recruited. All participants had equal training volume (13 h of training including one 60-min handball game per week). Exclusion criteria included any neuromuscular impairments and/or ankle, knee, hip trauma in the past 2 years and medication that might influence balance. Participants were also asked to avoid strenuous activity and the ingestion of alcohol or/and exciting substances 24 h before the experimental session. All participants voluntarily signed an informed consent form before starting the experiment, which was in accordance with the Helsinki Declaration. All procedures were approved by and performed in accordance with the relevant guidelines and regulations of the University of Pau and Adour Countries Ethics Committee.

### Balance control assessment

Participants were asked to stand barefoot and to sway as little as possible in a monopedal stance on their non-dominant leg for 25 s on a force platform (STABILOTEST TECHNO CONCEPT, Mane, France) which sampled the centre of foot pressure (COP) displacements at 40 Hz. The non-dominant leg (i.e. the supporting leg, which was determined as the leg which is not used to kick a ball) was chosen because handball is an asymmetric activity which requires frequent phases of monopedal stance on the non-dominant leg (e.g. while passing, jumping and shooting). A wobble board with a diameter of 40 cm and a height of 8 cm (Balance-board, SISSEL GmbH, Bad Dürkheim, Germany) could be placed on the force platform to generate instability. For accurate and similar feet positioning between all subjects, the foot was placed according to precise landmarks on the force platform and the wobble board. Subjects had the supporting leg extended and the other leg flexed with the big toe positioned at the level of the malleolus of the other leg (without any contact). They were also asked to cross the arms in front of the chest while touching the border of the clavicle with the forefinger. Two postural tasks were considered: a stable task (STA) where participants stood on stable ground with the eyes closed (while keeping their gaze in a straight-ahead direction) and an unstable task (UNSTA) where they stood on the wobble board with the eyes open (while looking at a fixed level target at a distance of 2 m). All postural tasks were performed with or without wearing compression garments (i.e., in the CG condition and the REF condition respectively). Two familiarization trials were performed for each postural task before data acquisition in order to avoid any learning effect^43^. COP surface area (S: 90% confidence ellipse), mean COP velocity along the medio-lateral (VX) and antero-posterior (VY) axes were calculated to characterize balance control; the lower these parameters, the more efficient the balance control^44^. In the CG condition, progressive calf compression sleeves composed of 69% Polyamide, 21% Elastane and 10% Yarn (BOOSTER Elite, BV SPORT, Saint Etienne, France) were worn by the participants. These CG provide a pressure that increases gradually from 13 mmHg at the ankle to 19 mmHg at the gastrocnemius. CG size was individualized according to guidelines of the manufacturers, based on participants’ height and calf circumference. Four sizes were used: M + , L + , XL + and XXL + , sized for 34–38 / > 175, 38–43 / > 175, 38–43 / > 192, 43–48/192 (calf circumference/height, in cm) respectively so as to accommodate all participants’ body shape.

### Statistical analysis

The two postural tasks (STA and UNSTA) were analyzed independently. The first step of the analysis consisted of evaluating the influence of CG with a standard whole group analysis. Since the data did not meet the assumption of normal distribution (tested with the Shapiro–Wilk test), non-parametric Wilcoxon signed rank tests were applied to compare each dependent variables (S, VX, VY) between the REF and CG conditions. A standardized principal component analysis (PCA) was also applied as a multivariate approach in order to conduce a more subtle descriptive analysis of variability between subjects, while identifying potential linear links between variables. COP parameters in the REF condition and the relative differences (RD) of each COP parameter between both conditions (RD = 100*[CG-REF]/REF) were used as input values in the PCA. RD is an easily interpretable descriptor, which limits the influence of the heterogeneity between participants in the REF condition and makes it easy to differentiate participants who benefit from CG wearing (negative value of RD) and those who do not (positive value of RD). Finally, a hierarchical cluster analysis (HCA)^45^ was used in a stepwise fashion on the basis of the principal component scores obtained from the PCA, to categorize the participants’ behaviours into subgroups with similar characteristics (i.e. with low or high COP values in the REF condition and low or high RD values)^27,28^. Statistical analyses were performed with R statistical software. The significance level was set at *P* < 0.05 and tendencies were reported when 0.05 ≤ *P* < 0.1.

## Abbreviations

CG: Compression garments
COP: Centre of foot pressure
HCA: Hierarchical cluster analysis
PC: Principal component
PCA: Principal component analysis
RD: Relative difference (RD = 100*[CG-REF]/REF)
REF: Reference
S: COP surface area
SD: Standard deviation
STA: Stable postural task with the eyes closed
UNSTA: Unstable postural task with the eyes open
VX: Mean COP velocity along the medio-lateral axis
VY: Mean COP velocity along the antero-posterior axis
